# Characteristics of diarrheagenic *Escherichia coli* among children under 5 years of age with acute diarrhea: a hospital based study

**DOI:** 10.1186/s12879-017-2936-1

**Published:** 2018-02-01

**Authors:** Yu Zhou, Xuhui Zhu, Hongyan Hou, Yanfang Lu, Jing Yu, Lie Mao, Liyan Mao, Ziyong Sun

**Affiliations:** Department of Laboratory Medicine, Tongji Hospital, Tongji Medical College, Huazhong University of Science and Technology, No. 1095 Jiefang Road, Wuhan, 430030 China

**Keywords:** Acute diarrhea, Diarrheagenic *Escherichia coli*, Antibiotic resistance, Atypical EPEC

## Abstract

**Background:**

Diarrhea is the leading infectious cause of childhood morbidity and mortality. Among bacterial agents, diarrheagenic *Escherichia coli* (DEC) is the major causal agent of childhood diarrhea in developing countries, particularly in children under the age of 5 years. Here, we performed a hospital-based prospective study to explore the pathotype distribution, epidemiological characteristics and antibiotic resistance patterns of DEC from < 5-year-old diarrheal children.

**Methods:**

Between August 2015 and September 2016, 684 stool samples were collected from children (< 5 years old) with acute diarrhea. All samples were cultured and identified by matrix-assisted laser desorption ionization time-of-flight mass spectrometry (MALDI-TOF MS) and biochemical tests. PCR was used for subtyping, and enteropathogenic *E. coli* (EPEC) isolates were identified simultaneously with serology. Furthermore, antimicrobial sensitivity tests and sequencing of antibiotic resistance-related genes were conducted.

**Results:**

DEC strains were identified in 7.9% of the 684 stool samples. Among them, the most commonly detected pathotype was EPEC (50.0% of DEC), of which 77.8% were classified as atypical EPEC (aEPEC). Age and seasonal distribution revealed that DEC tended to infect younger children and to occur in summer/autumn periods. Multidrug-resistant DEC isolates were 66.7%; resistance rates to ampicillin, co-trimoxazole, cefazolin, cefuroxime, cefotaxime, and ciprofloxacin were ≥ 50%. Among 5 carbapenem-resistant DEC, 60.0% were positive for carbapenemase genes (2 *blaNDM-1* and 1 *blaKPC-2*). Among 30 cephalosporin-resistant DEC, 93.3% were positive for extended-spectrum β-lactamase (ESBL) genes, with *blaTEM-1* and *blaCTX-M-55* being the most common types. However, no *gyrA* or *gyrB* genes were detected in 16 quinolone-resistant isolates. Notably, aEPEC, which has not received much attention before, also exhibited high rates of drug resistance (81.0%, 66.7%, and 14.3% for ampicillin, co-trimoxazole, and carbapenem resistance, respectively).

**Conclusions:**

EPEC was the most frequent DEC pathotype in acute diarrheal children, with aEPEC emerging as a dominant diarrheal agent in central China. Most DEC strains were multidrug-resistant, making even ciprofloxacin unsuitable for empiric treatment against DEC infection. Among carbapenem-resistant DEC strains, those harboring *blaNDM-1* and *blaKPC-2* were the main causal agents. *blaTEM-1* and *blaCTX-M-55* were the major genetic determinants associated with high levels of cephalosporin resistance.

**Electronic supplementary material:**

The online version of this article (10.1186/s12879-017-2936-1) contains supplementary material, which is available to authorized users.

## Background

Diarrheal disease is the leading infectious cause of childhood morbidity and mortality, most commonly occurring in sub-Saharan Africa and south Asia [1, 2]. China is one of the 15 high-incidence countries, with an annual estimate of 770 million episodes of childhood diarrhea [3]. Although many studies worldwide have reported rotavirus to be the primary cause of acute diarrhea in children, the role of bacteria in causing diarrhea appeared to differ depending on the geographical area [3–5]. Diarrheagenic *Escherichia coli* (DEC) is the leading cause of bacterial pediatric diarrhea in developing regions [6] and has been suggested to frequently occur in young children [7–9]. Results from a 5-year surveillance in China revealed DEC to be the most common bacterial pathogen among children younger than 5 years of age [4]. However, epidemiological data on DEC is still rare, particularly in China.

On the basis of specific virulence properties, DEC can be classified into 6 major categories: enteropathogenic *E. coli* (EPEC), enteroaggregative *E. coli* (EAEC), enterotoxigenic *E. coli* (ETEC), enteroinvasive *E. coli* (EIEC), Shiga toxin-producing *E. coli* (STEC), and diffusely adherent *E. coli* (DAEC) [10]. Among these, the first five 5 pathotypes have been frequently studied [11, 12]. In addition, EPEC can be divided into 2 subtypes according to the presence of bundle-forming pili, a fimbrial adhesin that is a virulence determinant of typical EPEC (tEPEC) but is absent from atypical EPEC (aEPEC) [13, 14]. While the pathogenic potential of aEPEC strains has been argued in the past, a study published in 2013 by the Global Enteric Multicenter Study indicated aEPEC to be the 5th most frequently detected pathogen in children aged 0–11 months who died of acute gastroenteritis [1]. To date, many severe outbreaks caused by DEC have been reported worldwide and have caused great losses [10].

A multicenter study conducted in China revealed notable differences in DEC categories between populations with different ages [15]. However, information about DEC strains isolated from children with acute diarrhea is sparse, because DEC is not routinely screened in most countries, including China [4]. Many studies have not addressed drug resistance tendency [15, 16], but the treatment for infectious agents of the *Enterobacteriaceae* family has been increasingly complicated by the emergence of strains resistant to most first-line antimicrobial agents in the last few decades [17, 18]. Here, we performed a hospital-based prospective study to explore the pathotype distribution and epidemiological characteristics of DEC from young children with diarrhea, as well as to reveal the grim situation of drug resistance in DEC strains.

## Methods

### Clinical definitions

The definition of diarrhea was as at least 3 abnormal appearance stools (loose, watery, mucus or bloody) in 24 h, with at least one of the following symptoms: nausea, vomiting, abdominal pain, or fever above 37.2 °C. The diarrhea which lasted 14 days or less at the time of presentation was defined as acute diarrhea, otherwise, was defined as persistent diarrhea. Persistent diarrheal Children were excluded from the present study [16].

### Study design and population

From August 2015 to September 2016, stool samples from acute diarrheal children under 5 years of age were collected at Tongji hospital (the largest teaching hospital in central China, which has more than 4000 beds and treats patients from the six surrounding provinces) [19]. All samples were collected under the parents’ or legal guardians’ permission. Demographic information for each patient, such as age, sex and clinical symptoms were collected.

### Identification of *E. coli*

All stool samples collected were processed by routine microbiological tests to identify. Briefly, MacConkey (Mac) agar were used to isolate the pathogens and incubated for 24 h at 37 °C. Three suspicious colonies with *E. coli* morphology (including lac + or lac-) were selected from Mac agar plates and all of them were identified by matrix-assisted laser desorption ionization time-of-flight mass spectrometry (MALDI-TOF MS) using the MALDI Biotyper (Bruker Daltonik GmbH, Leipzig, Germany). Biochemical tests were carried as supplements for *E. coli* identification. O157:H7 was screened by sorbitol-Mac.

### Molecular diagnostic methods for DEC

DEC was characterized by PCR as previously [19]: tEPEC (*eae* and *bfp*), aEPEC (*eae* or *bfp*), STEC (*eae* and *stx1* and/or *stx2*), ETEC (*elt* and/or *estIa* or *estIb*), EIEC (*virF* and *ipaH*) and EAEC (*aggR* and/or *pic* or *astA*). The PCR assay was carried out as follow**:** Boiling method was used for template DNA preparation. PCR was performed in 20 μl final volume. The reactions were run with the following cycling conditions: 94 °C for 2 min, 35 cycles of denaturation at 95 °C for 20 s, annealing at 60 °C for 30 s and primer extension at 72 °C for 45 s followed by a final extension at 72 °C for 5 min. EPEC CMCC44155, ETEC CMCC44815, EIEC CMCC44825 (from the National Institute for the Control of Pharmaceutical and Biological Products in China); EAEC serotype O42, STEC EDL933(from Chinese Center for Disease Control and Prevention) were served as the positive controls. *E. coli* DH5α, which lacks all the diarrhoeagenic genes, was used as a negative control.

#### Serotyping

EPEC serotyping and O157:H7 diagnosis were carried out by slide agglutination test using commercially available antisera (EPEC, O157:H7 antiserum from Ningbo Tianrun Bio-Pharmaceutical Co., Ltd., Zhejiang, China).

#### Antimicrobial sensitivity test

Antimicrobial susceptibilities were determined by the agar dilution method according to the Clinical and Laboratory Standards Institute (CLSI) Guidelines, 2015 [20]. All isolates of DEC were tested for their minimum inhibitory concentrations (MICs) of ampicillin, cefazolin, cefuroxime, cefotaxime, ceftazidime, cefepime, aztreonam, cefoxitin, ciprofloxacin, levofloxacin, gentamicin, amikacin, co-trimoxazole, piperacillin/tazobactam, imipenem and meropenem. Multi-drug resistance was defined as resistant to ≥ 3 antimicrobial categories. ATCC 25922, 35,218 and 27,853 were chosen as quality control strains. Results of antibiotic susceptibility were interpreted according to CLSI guidelines, 2015 [20].

### Molecular characterization of antibiotic resistance genes

Carbapenemase genes (*blaKPC-2, blaGES, blaIMP-4, blaVIM-1, blaNDM-1*and *blaOXA-48*) were screened in carbapenem-resistant DEC strains (MIC ≥ 4 ug/ml to imipenem or meropenem). Positive strains were further studied by sequencing. Strains showing significantly decreased susceptibility to ceftazidime or cefotaxime (MIC ≥ 32 ug/ml) were further studied by PCR amplification and sequencing of the extended-spectrum β-lactamase genes (ESBL genes, including *blaSHV, blaTEM* and *blaCTX-M*). Isolates showing high resistance to quinolones (MIC ≥ 32 ug/ml to ciprofloxacin or levofloxacin) were further tested for amino acid changes in the plasmid-mediated quinolone-resistant genes *gyrA* and *gyrB*, according to the methods reported by Yenkao et al. [21]. Quality control came from the strains identified by sequencing before. GenBank database (http://www.ncbi.nlm.nih.gov/) was employed to confirm the subtypes of antibiotic resistance genes.

## Statistical analysis

Using the software PASW Statistics 18.0 (IBM Corporation, New York), the chi-squared (*x*^*2*^) test was employed to determine the statistical significance of data. *P* value of < 0.05 was considered as statistically significant.

## Results

### Clinical features

Between August 2015 and September 2016, 684 stool samples were collected from diarrheal children under 5 years of age. In the population studied, most children (52.0%) were less than 24 months, 14.7% were 24–35 months, 16.9% were 36–47 months, and 16.4% were 48–59 months old, respectively. Boys accounted for 61.6% of the study population. Most patients were admitted in the summer (36.8%), followed by spring (23.5%), winter (21.6%), and autumn (18.0%). About half of the admitted children with acute diarrhea showed abdominal pain, followed by fever and vomiting (Details shown in Table 1).

**Table 1:** Basic information and clinical symptoms of the 684 children with acute diarrhea

Characteristic	Number (%)
Age(months)	
0–11	166(24.3%)
12–23	189(27.7%)
24–35	101(14.7%)
36–47	116(16.9%)
48–59	112(16.4%)
Sex	
Male	421(61.6%)
Female	263(38.4%)
Season	
Spring(January–March)	161(23.5%)
Summer(April–June)	252(36.8%)
Autumn(July–September)	123(18.0%)
Winter(October–December)	148(21.6%)
Clinical Symptoms	
Nausea	60(10.8%)
Vomiting	114(20.6%)
Abdominal pain	268(48.3%)
Fever(> 37.2 °C)	97(17.4%)
others	16(2.9%)

### Prevalence and epidemiological characteristics of DEC among infected children

**Fig. 1:**
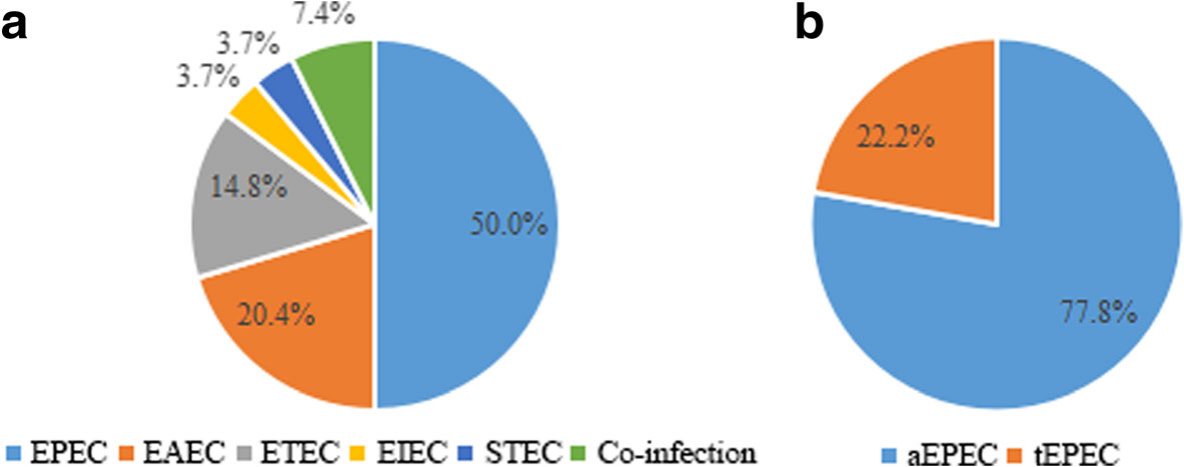
Distribution of DEC strains. **a** Pathotype distribution of 54 DEC strains. The colorful parts of pie chart showed the percentage of different DEC pathotypes. **b** Subtype distribution of 27 EPEC strains. The colorful parts of pie chart showed the percentage of different EPEC subtypes

Among 684 collected stool samples, 54 were positive for DEC (overall prevalence 7.9%). The most frequent pathotype was EPEC (50.0%), followed by EAEC (20.4%), ETEC (14.8%), EIEC (3.7%), and STEC (3.7%). Meanwhile, the remaining 7.4% cases were co-infected with more than one DEC pathotype (1 tEPEC and EAEC; 3 aEPEC and ETEC). Notably, among EPEC-infected cases, aEPEC accounted for 77.8%, while tEPEC accounted for just 22.2% (Fig. 1). Clinical data were available for 54 children with single or mixed DEC infection (shown in Additional files 1, 2 and 3). Fever was observed as the most frequent symptom (51.9%) among DEC-infected children, followed by vomiting (25.9%), abdominal pain (11.1%), bloody diarrhea (7.4%), and nausea (3.7%). Two children infected with non-O157:H7 STEC, both had bloody stools and showed severe symptoms of diarrhea. Worse symptoms were also discovered in children infected with more than one DEC pathotype.

**Fig. 2:**
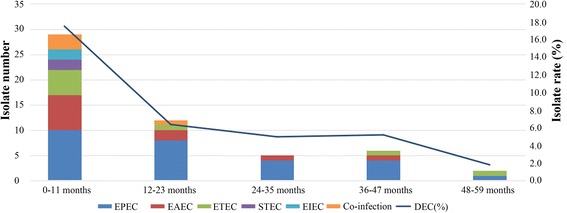
Age distribution of DEC strains among children with acute diarrhea (*n* = 54). The colorful bars showed the number of different DEC pathotypes in different age groups, broken line represented the isolate rate of DEC in different age groups

**Fig. 3:**
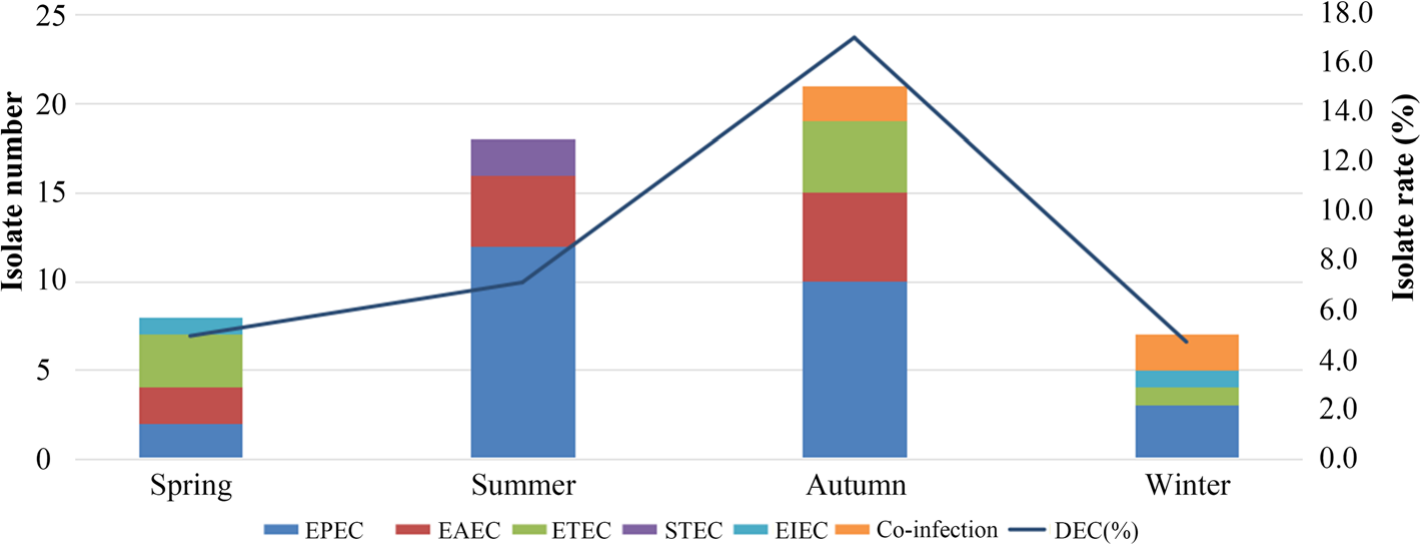
Seasonal distribution of DEC strains among children with acute diarrhea (*n* = 54). The colorful bars showed the number of different DEC pathotypes in different seasons, broken line represented the isolate rate of DEC in different seasons

Demography analysis showed that the younger the child was, the more prone to infection by DEC (chi-squared test, *P* < 0.001). When age stratification was done, the frequencies of DEC diarrheal episodes occurring in different age groups were 17.5% (0–11 months), 6.3% (12–23 months), 5.0% (24–35 months), 5.2% (36–47 months), and 1.8% (48–59 months) (Fig. 2). The isolate rate of DEC and the isolate number in subgroups by seasonality are shown in Fig. 3. The isolation rate of DEC showed a distinct seasonal variation, with a higher rate in the summer (7.1%) and autumn (17.1%) months (chi-squared test, *P* = 0.002). In addition, EPEC, the most dominant pathotype, tended to occur more in children less than 24 months of age (66.7%) and in the summer/autumn period (81.5%).

### Serotypes of EPEC and diagnosis of O157:H7

Among the 27 EPEC strains, only 11 (40.7%, 11/27) strains belonged to the classic EPEC serogroups, accounting for 33.3% (7/21) of aEPEC strains and 66.7% (4/6) of tEPEC strains. Furthermore, 22.2% strains belonged to serotype O86:k61; 11.1%, to O55:K59; and 7.4%, to O125:K70.

### Antibiotic resistance of DEC

**Fig. 4:**
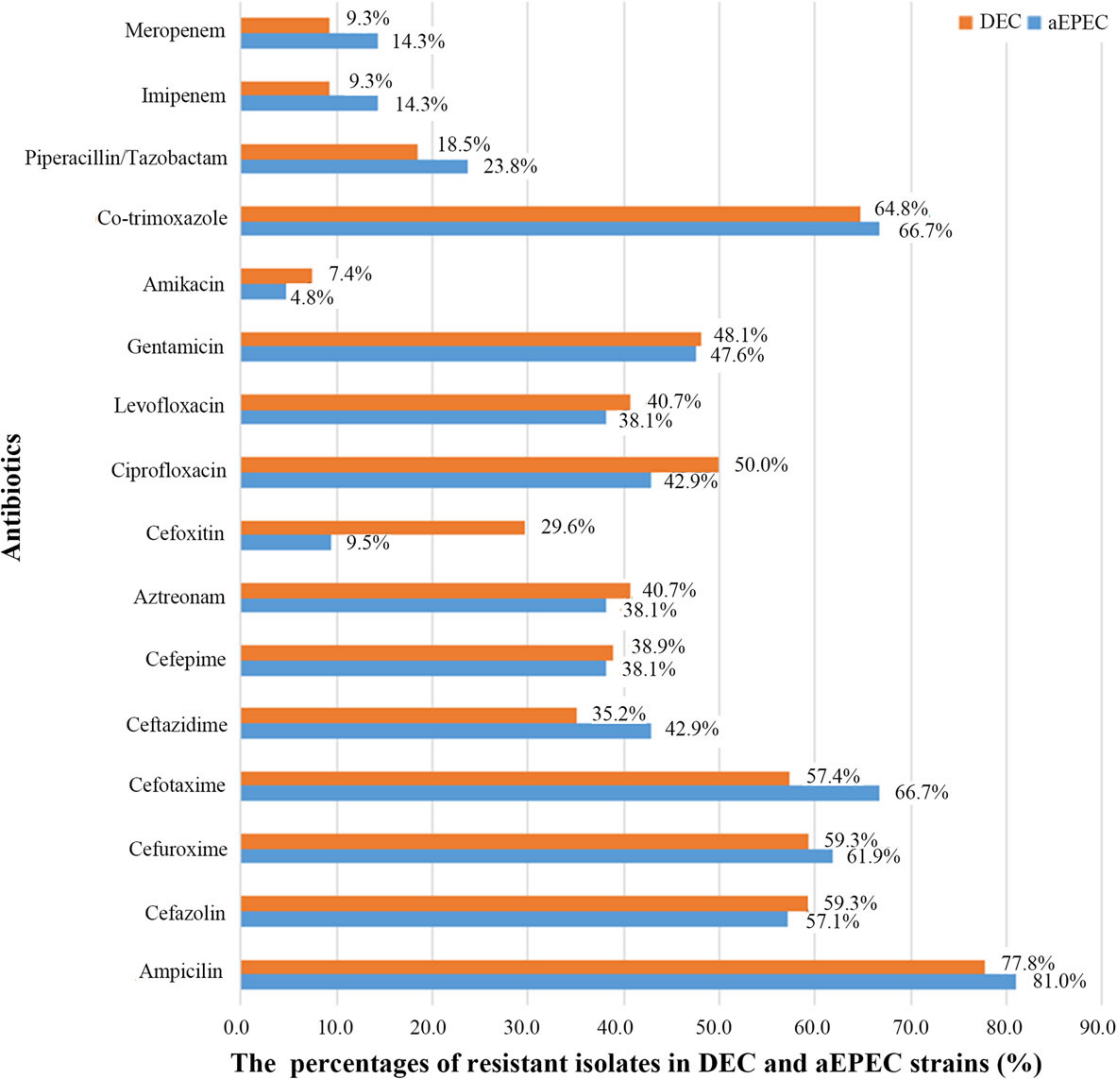
Drug resistance of DEC strains (*n* = 54) and aEPEC subtype (*n* = 21). The bars showed the percentages of resistant isolates of DEC and aEPEC subtype

The observed prevalence of resistance to 16 antibiotics amongst DEC are shown in Fig. 4. The highest resistance rate was detected for ampicillin (77.8%), followed by 64.8% for co-trimoxazole, 59.3% for cefazolin and cefuroxime; 57.4% for cefotaxime; 50.0% for ciprofloxacin; and ≥ 30% for gentamicin, aztreonam, levofloxacin, cefepime, and ceftazidime. For other antibiotics tested, cefoxitin, amikacin, piperacillin/tazobactam, imipenem, and meropenem showed efficacy against most DEC strains, with the resistance rate being < 30%. Although more than 90% DEC showed sensitivity to carbapenems, there was a 9.3% resistance rate. In addition, 36 (66.7%) isolates of DEC were multidrug-resistant, and 1 ETEC isolate showed resistance to all the antibiotics tested; this highlights the increasing trend of extensively drug-resistant bacteria. aEPEC, sharing high prevalence among DEC-infected patients, also had a high resistance rate, e.g., 81.0% for ampicillin, 66.7% for co-trimoxazole, and 14.3% for carbapenems (Fig. 4).

### Molecular analysis of antibiotic resistance genes among DEC strains

Among 5 (9.3%) carbapenem-resistant DEC strains, 3 (60%) isolates were positive for carbapenemase genes, with 2 harboring *blaNDM-1* and 1 harboring *blaKPC-2* (Table 2). One EIEC strain, harboring *blaKPC-2*, *blaCTX-M-65*, and *blaTEM-1*, showed resistance to most antibiotics tested, and was sensitive only to amikacin. Both *blaNDM-1*-positive strains showing high resistance to imipenem and meropenem (MIC ≥128 ug/ml) were aEPEC. There were 30 (55.5%) DEC isolates displaying high resistance to ceftazidime or cefotaxime (MIC ≥32 ug/ml). Among them, 93.3% were positive for ESBL genes, with *blaTEM-1* (43.3%) and *blaCTX-M-55* (33.3%) being the most common types. Positive rates of other ESBL genes detected were 20.0% for *blaCTX-M-15*, 10.0% each for *blaCTX-M-14* and *blaTEM-214*, and 3.3% each for *blaCTX-M-65* and *blaCTX-M-137*. *blaAmpC* genes and other carbapenemase genes were not observed. In addition, 16 (29.6%) isolates of DEC were found to be highly resistant to ciprofloxacin or levofloxacin (MIC ≥32 ug/ml), but no *gyrA* or *gyrB* genes were observed. Notably, aEPEC strains were found to harbor drug-resistance genes at a high rate. Among 3 carbapenemase-positive DEC strains, 2 strains positive for *blaNDM-1* were aEPEC, accounting for 66.7% of carbapenem-resistant aEPEC isolates. Meanwhile, 52.4% of cephalosporin-resistant aEPEC strains were positive for ESBL genes.

**Table 2:** Antibiotic resistance genes of 54 DEC strains

	Sex	Age(month)	Seasonality	Carbapenemase genes	ESBLs genes
EPEC(16/27)					
aEPEC(12/21)					
TJ1	Female	12–23	Autumn	*blaNDM-1*	*blaCTX-M-55*
TJ2	Male	0–11	Autumn		*blaTEM-1*
TJ3	Female	12–23	Summer		*blaCTX-M-14*
TJ4	Female	0–11	Winter		*blaTEM-1*
TJ5	Male	12–23	Winter		*blaCTX-M-55*
TJ6	Male	24–35	Autumn		*blaCTX-M-137;blaTEM-1*
TJ7	Male	24–35	Autumn		*blaCTX-M-14;blaTEM-1*
TJ8	Male	36–47	Autumn		*blaCTX-M-55;blaTEM-1*
TJ9	Male	36–47	Autumn		*blaCTX-M-15*
TJ10	Male	0–11	Summer		*blaTEM-214*
TJ11	Male	48–59	Winter	*blaNDM-1*	
TJ12	Male	0–11	Autumn		*blaTEM-1*
tEPEC(4/6)					
TJ13	Female	24–35	Summer		*blaCTX-M-15*
TJ14	Male	0–11	Summer		*blaTEM-1*
TJ15	Male	36–47	Autumn		*blaCTX-M-55*
TJ16	Female	24–35	Summer		*blaTEM-1*
EAEC(3/11)					
TJ17	Male	0–11	Autumn		*blaCTX-M-55;blaTEM-214*
TJ18	Female	0–11	Autumn		*blaCTX-M-55;blaTEM-214*
TJ19	Male	24–35	Autumn		*blaCTX-M-55;blaTEM-1*
ETEC(3/8)					
TJ20	Male	0–11	Autumn		*blaCTX-M-15*
TJ21	Male	36–47	Winter		*blaCTX-M-15*
TJ22	Male	48–59	Autumn		*blaCTX-M-55;blaTEM-1*
EIEC(1/2)					
TJ23	Male	0–11	Spring	*blaKPC-2*	*blaCTX-M-65;blaTEM-1*
STEC(2/2)					
TJ24	Female	0–11	Summer		*blaCTX-M-15;blaTEM-1*
TJ25	Male	0–11	Summer		*blaCTX-M-15*
Co-infection					
aEPEC + ETEC (2/3)					
TJ26	Male	0–11	Autumn		*blaCTX-M-55*
TJ27	Male	0–11	Autumn		*blaCTX-M-14*
tEPEC + EAEC(1/1)					
TJ28	Male	12–23	Winter		*blaCTX-M-55;blaTEM-1*

## Discussion

DEC is a public health risk for children, especially in developing countries [6]. In the present study, we evaluated the prevalence of DEC categories, epidemiological characteristics, and antibiotic resistance patterns among 684 young children with acute diarrhea in central China.

### Prevalence of DEC

The prevalence of DEC in our study was 7.9% (54/684), lower than reports from other developing countries [8, 22] but similar to the results of studies in China [4, 19, 23]. This suggests the influence of different regions in the distribution of DEC. The primary pathotypes were EPEC (54.0%) and EAEC (22.0%), corresponding with the results reported by Wang et al. [15]. EPEC, first named in 1995 by Neter et al. [24], has been described as the most frequent DEC pathotype in many developing countries [4, 25, 26]. Our study reflected that EPEC constituted 54.0% of DEC isolates, a little higher than that reported by Yu et al. [4], which supported the need for follow-up epidemiological studies in childhood diarrhea. EAEC strains have been associated with traveler’s diarrhea in both developing and industrialized countries [27, 28]. Studies carried out in Brazil and Mexico revealed EAEC to be the primary pathotype, with respective rates of 50% [12] and 52.1% [6]. In the present study, EAEC (20.5%) ranked as the second most common DEC associated with infected children, reflecting the difference in distribution across geographical areas. STEC, a subgroup of DEC strongly related to severe human illnesses [29], continued to be uncommon. However, the infection rate was a little higher (4.0%) in the present study compared to 0.4% in Beijing [23] and 0% in Shanghai [7]. Although no O157:H7 was observed, both STEC-infected children in this study presented with bloody stools. These findings should caution clinicians to carefully monitor the prevalence of STEC in children with severe diarrhea. Consistent with the findings of Patzi-Vargas et al. [25], EIEC was observed at a very low frequency (4.5%).

### Age and seasonal distribution of DEC infection cases

When age stratification was analyzed, the infection rate of DEC was found to decrease with age (*P* < 0.05), consistent with a report by Gomes et al. [13]. The lower prevalence in older children might be attributed to age-related immunity, which has also been observed in other studies [22, 25]. Generally speaking, EPEC is among the most important pathogens infecting children under 2 years of age in the developing country [6, 30]. Supporting this view, in the present study, 66.7% of EPEC infection cases were of children less than 24 months old. Seasonal variation was also found in DEC infection, especially in the EPEC group, which occurred most frequently in the late summer/early autumn period (*P* < 0.05). Similar seasonal patterns have also been observed in earlier studies [15, 25], indicating that DEC infection is strongly related to environmental factors such as temperature and humidity.

### High drug-resistant rate among DEC strains

The resistance rates of DEC to first-line therapeutic drugs were high, e.g., 77.8% to ampicillin and 64.8% to co-trimoxazole, higher than the rates we reported before [19, 31] but lower than those reported by Chen et al., where 91.8% DEC were resistant to ampicillin [32]. In this study, 36 (66.7%) DEC isolates were multidrug-resistant, comparable with 70.2% in the study by Chen et al. [32]. By molecular analysis, the ESBL genes *blaTEM-1* and *blaCTX-M-55* were the genetic determinants responsible for resistance to cephalosporins. ESBL-producing strains have been reported to be recently changing from the *blaTEM* or *blaSHV* type to *blaCTX-M* [18], which was consistent with our findings. *blaCTX-M-15* and *blaCTX-M-14* have been the most common cephalosporin-resistant genotypes isolated from humans [33], while *blaCTX-M-55* was the dominant type in the present study, followed by *blaCTX-M-15* and *blaCTX-M-14*. The molecular characterization of the isolates suggested that *blaCTX-M-55* is most closely related to *blaCTX-M-15*, with only a single amino acid substitution, indicating that *blaCTX-M-55* might be just a derivative of *blaCTX-M-15* [34]. In 2014, a nationwide investigation of ESBL- and AmpC-producing *E. coli* first reported that the incidence of *blaCTX-M-55* exceeded that of *blaCTX-M-15* in China [35], which warns of the prevalence of new variants of *blaCTX-M*. At present, carbapenems are the first option to treat ESBL-resistant strains, but the resistance rate of carbapenems (9.3%) presented a rising trend, with all the DEC showing sensitivity to imipenem in our earlier study [19, 31]. Carbapenem resistance in the *Enterobacteriaceae* is mainly attributed to the production of carbapenemases, the most common one being *blaKPC* [36] and the predominant one in China being *blaKPC-2* [37, 38]. In our study, both *blaKPC-2* and *blaNDM-1* were found to be associated with carbapenem resistance among DEC. *blaNDM-1*, first reported in 2008, demonstrated a current and pressing example of the rapidity with which it could disseminate globally [39]. Recently, *NDM-1*-producing *Klebsiella pneumoniae* was detected from the neonatal ward in our hospital [40], which reflected the spread of this resistance gene in China. In contrast with a published finding [19], mutations in the plasmid-mediated quinolone resistance genes *gyrA* and *gyr B* were not the major reason for resistance to quinolone antibiotics in the present study, suggesting that some other resistance mechanism might exist, e.g., overexpression of efflux pumps and decreased expression of outer membrane porins.

### aEPEC: The crucial pathotype in DEC-infected patients

Interestingly, aEPEC was found to be the crucial subtype of EPEC, accounting for 77.8% of EPEC strains and 42.0% of DEC strains. Although the association of aEPEC with diarrhea is still controversial [6], recent epidemiological studies have suggested an increasing identification of aEPEC in both developed and developing countries [28, 41], with some strains leading to diarrheal outbreaks [42]. A study from 13 developing countries showed that aEPEC isolates were responsible for 78% (131/169) of EPEC cases in children [43], which was similar with our study. Nonetheless, many existed studies lack the discrimination between tEPEC and aEPEC [4, 15, 16], which have made parallel contrast among different areas unavailable. Our study indicated aEPEC to be dominant and closely related with diarrhea among children, which has rarely been reported in China so far.

Hernandes et al. indicated that approximately 81% of the reported aEPEC strains did not belong to the classical EPEC serogroups, and 26.6% of them were O non-typeable [44]. In the present study, only 33.3% (7/21) of aEPEC strains belonged to the classical EPEC serogroups, indicating that serotyping might fail to detect many aEPEC strains. However, serotyping is still frequently used in many clinical laboratories, including in China. This raises concerns regarding correct diagnosis and might need to be addressed urgently.

aEPEC strains identified in Mexico were shown to possess high antibiotic resistance [6]. Similarly, aEPEC strains isolated from a food-poisoning outbreak in China were found to have high multidrug resistance, including high resistance to both quinolones and extended-spectrum cephalosporins [45]. In our study, aEPEC strains have developed resistance to many commonly used clinical drugs. Resistance rates of aEPEC to quinolones and extended-spectrum cephalosporins were all >30%. Furthermore, 2 *blaNDM-1* positive strains were both aEPEC strains. This implied that the drug resistance rate was high among aEPEC strains, warranting more attention to be focused on this critical aspect.

### Limitations

This study also had several limitations. Some DEC pathotypes were too few to demonstrate any association with age and seasonal patterns, which had been shown in other studies [15, 25]. Hence, larger sample size, extensive coverage area, and longer monitoring time are needed to yield an overall picture of DEC prevalence in childhood diarrhea. What is more, DAEC, another less well-defined pathotype, was not detected in our study for the difficulties in its identification and classification.

## Conclusion

Knowledge of the etiology of diarrhea is important for epidemiological surveillance. Our findings indicated EPEC to be the dominant pathotype in DEC infection in children under 5 years of age. Meanwhile, aEPEC, which is becoming the dominant subtype of EPEC, outnumbered tEPEC. The rising tendency of drug resistance among DEC strains was observed, including the relatively efficient drugs imipenem and meropenem. Most aEPEC strains, as well as DEC, also possessed high levels of antibiotic resistance. In isolates exhibiting cephalosporin resistance, the *blaTEM-1* and *blaCTX-M-55* genes were identified as the major resistance mechanisms. Meanwhile, *blaKPC-2* and *blaNDM-1* were the major carbapenemase genes associated with carbapenem resistance. These data call for further studies on DEC in children with diarrhea in China, as well as the need for continuous antimicrobial surveillance, with an emphasis on the rising prevalence of aEPEC.

## Additional files

Additional file 1: Clinical information of DEC infected children. (XLSX 27 kb)

Additional file 2: Raw data of MIC. (XLSX 24 kb)

Additional file 3: Sequencing for resistance genes. (DOCX 14 kb)

## Abbreviations

aEPEC: atypical EPEC
CLSI: Clinical and Laboratory Standards Institute
DEC: Diarrheagenic *Escherichia coli*
EAEC: enteroaggregative *E.coli*
EIEC: enteroinvasive *E.coli*
EPEC: enteropathogenic *E.coli*
ESBL: extended-spectrum β-lactamase
ETEC: enterotoxigenic *E.coli*
Mac: MacConkey
MALDI-TOF MS: matrix-assisted laser desorption ionization time-of-flight mass spectrometry
MIC: Minimal inhibitory concentration
STEC: Shiga toxin-producing *E.coli*
tEPEC: typical EPEC
