# Supplementary material for: Characteristics of diarrheagenic Escherichia coli among children under 5 years of age with acute diarrhea: a hospital based study
Source: BMC Infect Dis. 2018 Feb 1;18:63. doi: 10.1186/s12879-017-2936-1 (PMC5796495; doi:10.1186/s12879-017-2936-1)
Supplement: Supplementary file 3 — Sequencing for resistance genes. (DOCX 14 kb) [file 12879_2017_2936_MOESM3_ESM.docx]

*blaTEM:*

***blaTEM-1***: TTCCGGGGGGCCGCTATCCCTTTTTGCGGCATTTTGCCTTCCTGTTTTTGCTCACCCAGAAACGCTGGTGAAAGTAAAAGATGCTGAAGATCAGTTGGGTGCACGAGTGGGTTACATCGAACTGGATCTCAACAGCGGTAAGATCCTTGAGAGTTTTCGCCCCGAAGAACGTTTTCCAATGATGAGCACTTTTAAAGTTCTGCTATGTGGTGCGGTATTATCCCGTGTTGACGCCGGGCAAGAGCAACTCGGTCGCCGCATACACTATTCTCAGAATGACTTGGTTGAGTACTCACCAGTCACAGAAAAGCATCTTACGGATGGCATGACAGTAAGAGAATTATGCAGTGCTGCCATAACCATGAGTGATAACACTGCTGCCAACTTACTTCTGACAACGATCGGAGGACCGAAGGAGCTAACCGCTTTTTTGCACAACATGGGGGATCATGTAACTCGCCTTGATCGTTGGGAACCGGAGCTGAATGAAGCCATACCAAACGACGAGCGTGACACCACGATGCCTGCAGCAATGGCAACAACGTTGCGCAAACTATTAACTGGCGAACTACTTACTCTAGCTTCCCGGCAACAATTAATAGACTGGATGGAGGCGGATAAAGTTGCAGGACCACTTCTGCGCTCGGCCCTTCCGGCTGGCTGGTTTATTGCTGATAAATCTGGAGCCGGTGAGCGTGGGTCTCGCGGTATCATTGCAGCACTGGGGCCAGATGGTAAGCCCTCCCGTATCGTAGTTATCTACACGACGGGGAGTCAGAGTTTTTTGTTTGA

***blaTEM-214:***

CCTGCGGGCCGCGCTTTCCCTTTTTGCGGCATTTTGCCTTCCTGTTTTTGCTCACCCAGAAACGCTGGTGAAAGTAAAAGATGCTGAAGATCAGTTGGGTGCACGAGTGGGTTACATCGAACTGGATCTCAACAGCGGTAAGATCCTTGAGAGTTTTCGCCCCGAAGAACGTTTTCCAATGATGAGCACTTTTAAAGTTCTGCTATGTGGTGCGGTATTATCCCGTGTTGACGCCGGGCAAGAGCAACTCGGTCGCCGCATACACTATTCTCAGAATGACTTGGTTGAGTACTCACCAGTCACAGAAAAGCATCTTACGGATGGCATGACAGTAAGAGAATTATGCAGTGCTGCCATAACCATGAGTGATAACACTGCTGCCAACTTACTTCTGACAACGATCGGAGGACCGAAGGAGCTAACCGCTTTTTTGCACAACATGGGGGATCATGTAACTCGCCTTGATCGTTGGGAACCGGAGCTGAATGAAGCCATACCAAACGACGAGCGTGACACCACGATGCCTGCAGCAATGGCAACAACGTTGCGCAAACTATTAACTGGCGAACTACTTACTCTAGCTTCCCGGCAACAATTAATAGACTGGATGGAGGCGGATAAAGTTGCAGGACCACTTCTGCGCTCGGCCCTTCCGGCTGGCTGGTTTATTGCTGATAAATCTGGAGCCGGTGAGCGTGGGTCTCGCGGTATCATTGCAGCACTGGGGCCAGATGGTAAGCCCTCCCGTATCGTAGTTATCTACACGACGGGATCAGATTTTTGTGTGTGTGG

*blaCTX-M*

***blaCTX-M-55:*** TTCATTTTTGGATATCCGGTGGTGGTGCCATAGCCACCGCTGCCGGTTTTATCCCCCACAACCCAGGAAGCAGGCAGTCCAGCCTGAATGCTCGCTGCACCGGTGGTATTGCCTTTCATCCATGTCACCAGCTGCGCCCGTTGGCTGTCGCCCAATGCTTTACCCAGCGTCAGATTCCGCAGAGTTTGCGCCATTGCCCGAGGTGAAGTGGTATCACGCGGATCGCCCGGAATGGCGGTGTTTAACGTCGGCTCGGTACGGTCGAGACGGAACGTTTCGTCTCCCAGCTGTCGGGCGAACGCGGTGACGCTAGCCGGGCCGCCAACGTGAGCAATCAGCTTATTCATCGCCACGTTATCGCTGTACTGTAGCGCGGCCGCGCTAAGCTCAGCCAGTGACATCGTCCCATTGACGTGCTTTTCCGCAATCGGATTATAGTTAACAAGGTCAGATTTTTTGATCTCAACTCGCTGATTTAACAGATTCGGTTCGCTTTCACTTTTCTTCAGCACCGCGGCCACGGCCATCACTTTACTGGTATGGCAATTTCCCAAAAAA

***blaCTX-M-15***: GGAATTTTCCCGGGGTGTGCCATAGCCACCGCTGCCGGTTTTATCCCCCACAACCCAGGAAGCAGGCAGTCCAGCCTGAATGCTCGCTGCACCGGTGGTATTGCCTTTCATCCATGTCACCAGCTGCGCCCGTTGGCTGTCGCCCAATGCTTTACCCAGCGTCAGATTCCGCAGAGTTTGCGCCATTGCCCGAGGTGAAGTGGTATCACGCGGATCGCCCGGAATGGCGGTGTTTAACGTCGGCTCGGTACGGTCGAGACGGAACGTTTCGTCTCCCAGCTGTCGGGCGAACGCGGTGACGCTAGCCGGGCCGCCAACGTGAGCAATCAGCTTATTCATCGCCACGTTATCGCTGTACTGTAGCGCGGCCGCGCTAAGCTCAGCCAGTGACATCGTCCCATTGACGTGCTTTTCCGCAATCGGATTATAGTTAACAAGGTCAGATTTTTTGATCTCAACTCGCTGATTTAACAGATTCGGTTCGCTTTCACTTTTCTTCAGCACCGCGGCCGCGGCCATCACTTTACTGGTACTGCCACATCGCAAAAATGGGTTTAAGGGA

***blaCTX-M-14*:** TTTTTCTTTGGGGGTCCATAGTCGCCGCTGCCGGTCTTATCACCCACAGTCCACGACGTCGGTAAGCCGGCCCGAATGCTGGCTGCGCCGGTCGTATTGCCTTTGAGCCACGTCACCAACTGCGCCCGCTGGGTTTCGCCCAGCGCATGACCCAGCGTAAGCTGACGCAACGTCTGCGCCATCGCCCGCGGCGTGGTGGTGTCTCTCGGGTCGCCGGGAATGGCGGTATTCAGCGTAGGTTCAGTGCGATCCAGACGAAACGTCTCATCGCCGATCGCGCGGGCAAAAGCCGTCACGCCTCCCGGGCCACCGAGCTGGGCAATCAATTTGTTCATGGCGGTATTGTCGCTGTACTGCAACGCGGCCGCGCTCAGTTCTGCCAGCGTCATTGTGCCGTTGACGTGTTTTTCGGCAATCGGATTGTAGTTAACCAGATCGGCAGGCTTGATCTCGACAGGCTGATTAAGCAGCTGCTTTTGCGTTTCACTCTGCTTAAGCACCGCCGCGGCCGCCATAACTTTACTGGTATGCCCAATCCGCAAAAAAAA

***blaCTX-M-137*:**

GGGCGGTTTTTTTGGGAAATTGTCATACCAGTAAAGTTATGGCGGCCGCGGCGGTGCTTAAGCAGAGTGAAACGCAAAAGCAGCTGCTTAATCAGCCTGTCGAGATCAAGCCTGCCGATCTGGTTAACTACAATCCGATTGCCGAAAAACACGTCAACGGCACAATGACGCTGGCAGAACTGAGCGCGGCCGCGTTGCAGTACAGCGACAATACCGCCATGAACAAATTGATTGCCCAGCTCGGTGGCCCGGGAGGCGTGACGGCTTTTGCCCGCGCGATCGGCGATGAGACGTTTCGTCTGGATCGCACTGAACCTACGCTGAATACCGCCATTCCCGGCGACCCGAGAGACACCACCACGCCGCGGGCGATGGCGCAAACTCTGCGGAATCTGACGCTGGGTAAAGCATTGGGCGACAGCCAACGGGCGCAGCTGGTGACATGGATGAAAGGCAATACCACCGGTGCAGCGAGCATTCAGGCTGGACTGCCTGCTTCCTGGGTTGTGGGGGATAAAACCGGCAGCGGTGGCTATGGCACCCAACCGGAAAAAACCA

***blaCTX-M-65***: GCTTGGATATTTGGTTGGGGTGCCATAGTCGCCGCTGCCGGTCTTATCACCCACAGTCCACGACGTCGGTAAGCCGGCCCGAATGCTGGCTGCGCCGGTCGTATTGCCTTTGAGCCACGTCACCAACTGCGCCCGCTGGGTTTCGCCCAGCGCATGACCCAGCGTAAGCTGACGCAACGTCTGCGCCATCGCCCGCGGCGTGGTGGTGTCTCTCGGGTCGCCGGGAATGGCGGTATTCAGCGTAGGTTCAGTGCGATCCAGACGAAACGTCTCATCGCCGATCGCGCGGGCAAAAGCCGTCACGCCTCCCGGGCCACCGAGCTGGGCAATCAATTTGTTCATGGCGGTATTGTCGCTGTACTGCAACGCGGCCGCGCTCAGTTCTGCCAGCGTCATTGTGCCGTTGACGTGTTTTTCGGCAATCGGATTGTAGTTAACCAGATCGGCAGGCTTGATCTCGACAGGCTGATTAAGCAGCTGCTTTTGCGTTTCACTCTGCTTAAGCACCGCCGCGACCGCCATAACTTTACTGGTACTGCCCATTCCCCAAAAACA

***blaKPC-2***: ATTATGTCACCGGGTATGCCGTCTAGTTCTGCTGTCTTGTCTCTCATGGCCGCTGGCTGGCTTTTCTGCCACCGCGCTGACCAACCTCGTCGCGGAACCATTCGCTAAACTCGAACAGGACTTTGGCGGCTCCATCGGTGTGTACGCGATGGATACCGGCTCAGGCGCAACTGTAAGTTACCGCGCTGAGGAGCGCTTCCCACTGTGCAGCTCATTCAAGGGCTTTCTTGCTGCCGCTGTGCTGGCTCGCAGCCAGCAGCAGGCCGGCTTGCTGGACACACCCATCCGTTACGGCAAAAATGCGCTGGTTCCGTGGTCACCCATCTCGGAAAAATATCTGACAACAGGCATGACGGTGGCGGAGCTGTCCGCGGCCGCCGTGCAATACAGTGATAACGCCGCCGCCAATTTGTTGCTGAAGGAGTTGGGCGGCCCGGCCGGGCTGACGGCCTTCATGCGCTCTATCGGCGATACCACGTTCCGTCTGGACCGCTGGGAGCTGGAGCTGAACTCCGCCATCCCAGGCGATGCGCGCGATACCTCATCGCCGCGCGCCGTGACGGAAAGCTTACAAAAACTGACACTGGGCTCTGCACTGGCTGCGCCGCAGCGGCAGCAGTTTGTTGATTGGCTAAAGGGAAACACGACCGGCAACCACCGCATCCGCGCGGCGGTGCCGGCAGACTGGGCAGTCGGAGACAAAACCGGAACCTGCGGAGTGTATGGCACGGCAAATGACTATGCCGTCGTCTGGCCCACTGGGCGCGCACCTATTGTGTTGGCCGTCTACACCCGGGCGCCTAACAAGGATGACAAGCACAGCGAGGCCGTCATCGCCGCTGCGGCTAGACTCGCGCTCGAGGGATTGGGCGTCAACGGGCAGTAGGGGTTCTGAAAAAAAAAAAAAAAGAA

***blaNDM-1:*** TATTGGCATAAGTCGCAATCCCCGCCGCATGCAGCGCGTCCATACCGCCCATCTTGTCCTGATGCGCGTGAGTCACCACCGCCAGCGCGACCGGCAGGTTGATCTCCTGCTTGATCCAGTTGAGGATCTGGGCGGTCTGGTCATCGGTCCAGGCGGTATCGACCAACAGCACGCGGCCGCCATCCCTGACGATCAAACCGTTGGAAG
